# Closed reduction and percutaneous pinning vs open reduction and internal fixation in pediatric lateral condylar humerus fractures displaced by > 4 mm: an observational cross-sectional study

**DOI:** 10.1186/s12891-021-04880-8

**Published:** 2021-11-25

**Authors:** Li-wei Xie, Zhi-qiang Deng, Ren-huan Zhao, Juan Wang, Xin Liu, Ying Zhou, Hui Zhang

**Affiliations:** 1 Department of Pediatric Orthopedics, Sichuan Provincial Orthopedics Hospital, Sichuan Province, Chengdu, China; 2 Department of Geriatrics, Chengdu Shuang-nan Hospital, Chengdu, Sichuan China; 3 Department of Orthopaedic Surgery, West China Hospital, Sichuan University, Chengdu, Sichuan China

**Keywords:** Lateral condylar humerus fractures, Children, Closed reduction and percutaneous pinning, Open reduction and internal fixation

## Abstract

**Background:**

Although open reduction and internal fixation (ORIF) is recommended for lateral condylar humerus fractures (LCHFs) displaced by > 4 mm, several studies have reported the use of closed reduction and percutaneous pinning (CRPP) to treat LCHFs with significant displacement. However, little is known about the clinical differences between these two surgical techniques. This study aimed to compare the therapeutic effects of CRPP and ORIF in treating LCHFs displaced by > 4 mm.

**Methods:**

We retrospectively reviewed pediatric LCHFs displaced by > 4 mm treated with either CRPP or ORIF at our center from June 2019 to October 2020. Song and Milch fracture classifications were used. Variables such as age at injury, sex, side injured, fracture displacement, fracture type, operating time, postoperative treatment, and complications were compared between the two techniques.

**Results:**

One hundred twenty LCHFs met inclusion criteria. There were 36 Milch type I and 84 type II LCHFs, and 69 Song stage 4 and 51 stage 5 LCHFs. CRPP was performed in 45 cases and ORIF in 75 cases. No differences were found in age, sex, side injured, preoperative displacement, postoperative displacement, and length of immobilization between the CRPP and ORIF groups. There was a difference between operation time and pin duration. The CRPP group had shorter operation times and pin duration, and required no additional operations to remove internal pins. The average follow-up duration was 13.9 months. All patients achieved fracture union, and no complications such as infection, nonunion, delayed union, osteonecrosis, fishtail deformity, cubitus varus or valgus, or pain were recorded during follow-up. Bone spurs, lateral prominences, and decreased carrying angle were common complications in all groups. No obvious cubitus varus was observed. Unaesthetic scars were only observed in the ORIF groups. No differences in range of motion or elbow function was found among the different therapies.

**Conclusions:**

Both CRPP and ORIF can achieve satisfactory clinical outcomes in treating LCHFs displaced by > 4 mm. No differences were found in complications or prognoses between the two groups. However, CRPP shows some advantages over ORIF, like less invasive surgery, no obvious scarring, and no need for secondary surgery with anesthesia for pin removal.

## Background

Lateral condylar humerus fractures (LCHFs) are the second most common distal humeral fractures in children, accounting for approximately 17% of pediatric distal humeral fractures [1, 2]. Traditionally, LCHFs that are displaced by > 2 mm are treated with open reduction and internal fixation (ORIF) to ensure that anatomic reduction of this intra-articular fracture is achieved [1, 2]. Closed reduction and percutaneous pinning (CRPP) has been recommended for LCHFs with displacement between 2 mm and 4 mm because of its advantages over ORIF including less soft tissue dissection; lower risk of vessel damage, non-union, and osteonecrosis of the distal humerus epiphysis; shorter operating times; avoidance of an open incision and an unaesthetic scar; avoidance of second anesthesia and operation for hardware removal [1–10].

For LCHFs displaced by > 4 mm, direct ORIF is the treatment strategy adopted by most surgeons. Few studies have reported the use of CRPP in LCHFs displaced by > 4 mm. Song introduced a new classification (Table 1) and reported a CRPP success rate as high as 75% (18/24) with favorable results in LCHFs displaced by > 4 mm, even in cases with total displacement [11, 12]. Justus et al. reported a series of 172 patients with surgically treated LCHFs, where 18% (31) of the cases, including 13 Song stage 4 LCHFs and 4 Song stage 5 LCHFs, were treated with CRPP at the surgeons’ discretion [13]. However, Ramo et al. could not replicate the success of CRPP in Song stage 5 LCHFs in their series [14]. We previously reported a series of 46 LCHFs displaced by > 2 mm at our institute, where CRPP was attempted with an approximate 75% success rate, even in Song stage 5 cases [15]. These results illustrate the potential promise of the CRPP technique [13–15].

**Table 1:** The Song classification of pediatric lateral condylar humerus fractures

Stage	Degree of Displacement	Fracture Pattern	Radiograph Views Used as Basis	Stability
1	≤2 mm	Limited fracture line within the metaphysis	All 4 views	Stable
2	≤2 mm	Fracture line extends to the epiphyseal articular cartilage with a Lateral gap	All 4 views	Indefinable
3	≤2 mm	Gap as wide laterally as medially	Any of 4 views	Unstable
4	>2 mm	Without rotation of fragment	Any of 4 views	Unstable
5	>2 mm	With rotation of fragment	Any of 4 views	Unstable

Modified from: Song KS, Kang CH, Min BW, Bae KC, Cho CH, Lee JH. Closed reduction and internal fixation of displaced unstable lateral condylar fractures of the humerus in children. J Bone Joint Surg Am*.* 2008;90(12):2673–81 [11]

To date, few studies have compared the clinical outcomes of CRPP versus ORIF in treating LCHFs. Most studies have focused on LCHFs with minimal to moderate displacement (2 mm–4 mm), with no reported differences in outcomes between the two techniques [7, 13, 14]. However, few studies have compared the therapeutic effect of CRPP versus ORIF in LCHFs displaced by > 4 mm [13]. Therefore, we conducted the present study to compare the therapeutic benefits of CRPP versus ORIF in the treatment of LCHFs displaced by > 4 mm.

## Methods

### Patients

This observational cross-sectional record-based study retrospectively reviewed all LCHFs treated at our pediatric orthopedic center between June 2019 and October 2020. Inclusion criteria were as follows: age below 14 years, fresh closed fractures without additional injuries, fractures displaced by > 4 mm, the use of surgical treatment, and the presence of complete data. Exclusion criteria were as follows: age > 14 years, open fractures, old fractures, fractures displaced by < 4 mm or with additional injuries, the use of conservative treatment, pre-existing health conditions, and the presence of incomplete data. Finally, patients were divided into three groups: Group 1: cases treated with CRPP; Group 2: cases failed CRPP and converted to ORIF; Group 3: cases treated directly with ORIF. Furthermore, Group 1 was classified as CRPP group; Group 2 and Group 3 were classified as ORIF group. All cases were classified according to the Song and Milch classifications [11, 16].

### Surgical technique

CRPP was attempted in the treatment of LCHFs in Groups 1 and 2 by one surgical team, following methods previously described by Song et al. and Xie et al. ORIF was performed where adequate closed reduction (displacement of < 2 mm) could not be achieved. Intraoperative arthrography was used to confirm the congruence of the articular surface. LCHFs in Group 3 were treated by another surgical team that used the traditional lateral approach.

### Postoperative treatment and follow-up

Postoperatively, the affected arm was immobilized at 70° of elbow flexion with a posterior long-arm cast, with cast removal scheduled 4 weeks postoperatively depending on the extent of fracture union. In the CRPP group, the pins were removed 1 week post cast removal. Functional exercises were prescribed following cast or pin removal and performed by a rehabilitation team. Complications were recorded during follow-up. Elbow joint function was graded according to the Hardacre criteria [17].

### Statistical analysis

SPSS 26.0 (IBM Corp., Armonk, NY, USA) was used for statistical analysis. Continuous variables were analyzed using the independent-samples t-test, expressed as mean and standard deviation (mean ± SD). Count variables were analyzed by chi-square or Fisher’s exact test and expressed as numbers. Statistical significance was set at *P* < 0.05.

## Results

We treated 246 patients with LCHFs during the study period. The following cases were excluded: 46 cases treated conservatively, 19 old fractures, 22 cases associated with other injuries (10 ipsilateral proximal ulnar fractures; 6 ipsilateral coronoid process fractures; 4 ipsilateral elbow joint dislocations; 2 ipsilateral distal ulna and radius fractures), one patient with cerebral palsy, 8 cases with incomplete data, and 30 LCHFs with displacement < 4 mm. The remaining 120 LCHFs met the inclusion criteria for the present study. In total, there were 79 boys and 41 girls, with an average age of 5.3 ± 2.3 years. Thirty-six were Milch type I and 84 were type II LCHFs, and 69 were Song stage 4, and 51 were stage 5 LCHFs. Among these, there were 45 cases in Group 1, 13 cases in Group 2, and 62 cases in Group 3 (Table 2).

**Table 2:** Patient demographics

Variables	Group 1 (***n*** = 45)	Group 2 (***n*** = 13)	Group 3 (***n*** = 62)	Overall (***n*** = 120)	***P*** value
Age (years)	5.3 ± 2.5	5.7 ± 2.5	5.1 ± 2.2	5.3 ± 2.3	0.65
Sex					0.27
Male	27	11	41	79	
Female	18	2	21	41	
Side injured					0.75
Right	22	5	31	58	
Left	23	8	31	62	
Pre-op displacement (mm)	11.3 ± 6.1	10.1 ± 4.4	10.2 ± 4.4	10.6 ± 5.1	0.53
Post-op displacement (mm)	1.6 ± 0.5	1.3 ± 0.7	1.3 ± 0.8	1.4 ± 0.7	0.25
Operation Time (mins)	38.8 ± 11.8	70.2 ± 8.9	49.7 ± 7.2	48.8 ± 13.1	<0.001
Immobilization (weeks)	4.7 ± 1.0	4.4 ± 0.7	4.6 ± 0.6	4.6 ± 0.8	0.34
Pin duration (weeks)	5.6 ± 0.8	15.7 ± 2.6	15.9 ± 2.7	12.0 ± 5.5	<0.001
Follow-up (months)	14.2 ± 4.4	13.1 ± 3.3	13.9 ± 3.3	13.9 ± 3.7	0.66
Milch type					0.009
I	8	7	21	36	
II	37	6	41	84	
Song stage					0.65
4	24	6	39	69	
5	21	7	23	51	

*Pre-op* Pre-operative, *Post-op* Post-operative. Statistical significance was set at *P* < 0.05

No differences were found among the three groups in age, sex, side injured, preoperative displacement, postoperative displacement, or length of immobilization (*P* > 0.05). However, there was a significant difference in operative times and pin duration. The CRPP group had shorter operative times and pin duration than those in the other groups (*P* < 0.001), and needed no additional operations to remove the internal pins (Table 2). In the CRPP group, the Milch type II LCHFs achieved a higher success rate (*P* = 0.009, < 0.01) when using the Milch classification; when using the Song classification, no difference in CRPP success rate was found between Song stage 4 and 5 LCHFs (*P* = 0.65, > 0.05) (Table 3).

**Table 3:** Success rate of CRPP between different fracture types

Types of LCHFs	Group 1 (***n*** = 45)	Group 2 (***n*** = 13)	Sum (***n*** = 58)	***P*** value
Milch type				0.009
I	8	7	15	
II	37	6	43	
Song stage				0.65
4	24	6	30	
5	21	7	28	
Sum	45	13	58	

Statistical significance was set at *P* < 0.05

**Fig. 1:**
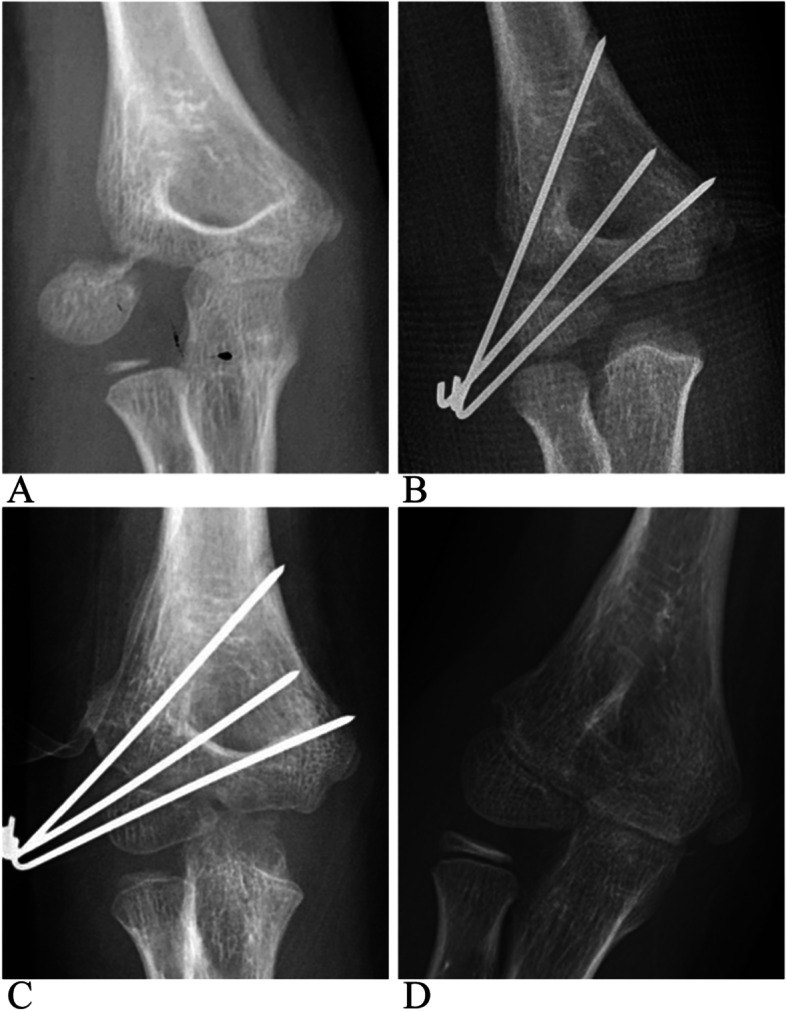
A Song stage 5 and Milch typeIILCHF of an 11 years old boy. **A** Pre-operative anterior-posterior X-ray. **B** Post-operative X-ray after CRPP. **C** X-ray 6 weeks post surgery. **D** X-ray 11 months post surgery

The average follow-up duration was 13.9 months (range: 9–25 months). All patients achieved fracture union (Fig. 1). Complications such as infection, nonunion, delayed union, osteonecrosis, fishtail deformity, cubitus varus or valgus, or pain were not recorded during follow-up, and no differences were found among any of the groups in these aspects. Bone spurs were observed in almost all cases on radiographs, but lateral prominences were only found in some cases. No differences were found in the incidence of lateral prominences in the different groups (*P* = 0.38, > 0.05). Cases with a slightly decreased carrying angle (< 5°) were found in all groups, with no differences in incidence among the groups (*P* = 0.96, > 0.05). No obvious cubitus varus was observed in any of the cases. Unaesthetic scars were found in 21 cases treated with ORIF, and underwent scar repair during hardware removal. All patients who underwent ORIF required additional anesthesia and surgery for hardware removal. However, all cases achieved satisfactory post-operative range of motion and elbow function, with no differences found among the different therapies (Table 4).

**Table 4:** Complications and functional outcomes

Complications	Group 1 (***n*** = 45)	Group 2 (***n*** = 13)	Group 3 (***n*** = 62)	Overall (***n*** = 120)	***P*** value
Infection	None	None	None	None	/
Delayed union	None	None	None	None	/
Nonunion	None	None	None	None	/
Osteonecrosis	None	None	None	None	/
Fishtail deformity	None	None	None	None	/
Cubitus varus	None	None	None	None	/
Cubitus valgus	None	None	None	None	/
Pain	None	None	None	None	/
Bone spur	45/45	13/13	62/62	120/120	/
Lateral prominence	4/45	3/13	9/62	120/120	0.38
Decreased carrying angle	3/45	1/13	5/62	9/120	0.96
Unaesthetic scar	0/45	4/13	17/62	21/120	<0.001
Additional operation	0/45	13/13	62/62	75/120	<0.001
Range of Motion (°)					
Extension	6.8 ± 5.2	6.2 ± 2.2	5.8 ± 2.0	6.2 ± 3.6	0.41
Flexion	133.2 ± 2.4	132.7 ± 2.6	132.7 ± 3.2	132.9 ± 2.9	0.60
Arc	140.1 ± 4.7	140.7 ± 4.5	138.7 ± 3.2	139.5 ± 4.0	0.10
Hardacre criteria					0.07
Excellent	42	12	57	111	
Good	3	1	5	9	
Poor	None	None	None	None	

Statistical significance was set at *P* < 0.05

## Discussion

The present study aimed to compare the therapeutic benefits of CRPP versus ORIF in the treatment of LCHFs displaced by > 4 mm. The results demonstrated no significant differences in clinical efficacy between CRPP and ORIF in treating LCHFs displaced by > 4 mm. Furthermore, our results showed that CRPP possesses several advantages over ORIF, including reduced invasiveness, shorter operation times, no obvious scars, and no need for secondary surgery with anesthesia for pin removal.

We focused on LCHFs displaced by > 4 mm because consensus has been reached regarding the treatment of LCHFs displaced between 2 mm and 4 mm. For such cases, it is recommended that CRPP should be attempted first, and if this fails, ORIF should be performed [1, 2, 9]. However, there is no consensus about the optimal treatment for LCHFs displaced by > 4 mm: most clinicians advocate for the use of ORIF, and only a few recommend CRPP [1–15].

Song et al. introduced a new classification and treatment strategy for LCHFs. According to their algorithm, all unstable LCHFs displaced by > 2 mm were first treated with CRPP. If a fracture gap of less than 2 mm could not be achieved after closed reduction, ORIF would then be performed. Their preliminary results showed that 76% (13/17) of stage 3 fractures, 75% (30/40) of stage 4 fractures, and 50% (3/6) of stage 5 fractures were successfully treated with CRPP [11]. A later study reported a 75% (18/24) success rate for stage 5 fractures. The follow-up period found excellent or good clinical outcomes in all cases with minor complications such as asymptomatic bone spurs at the lateral distal humerus [12]. Therefore, they reported that CRPP could be used to successfully treat LCHFs with greater displacement, even in completely displaced and rotated cases [11, 12]. We carefully studied the CRPP technique introduced by Song et al. and attempted it in a consecutive series. We achieved a 78% (36/46) overall success rate in LCHFs displaced by > 2 mm, which was comparable with Song’s results. We successfully treated all Song stages 2 and 3 cases, 76% (19/25) of Song stage 4 cases, and 78% (14/18) of Song stage 5 cases with CRPP. All patients achieved fracture union. The final follow-up found no dysfunction or major complications [15].

**Fig. 2:**
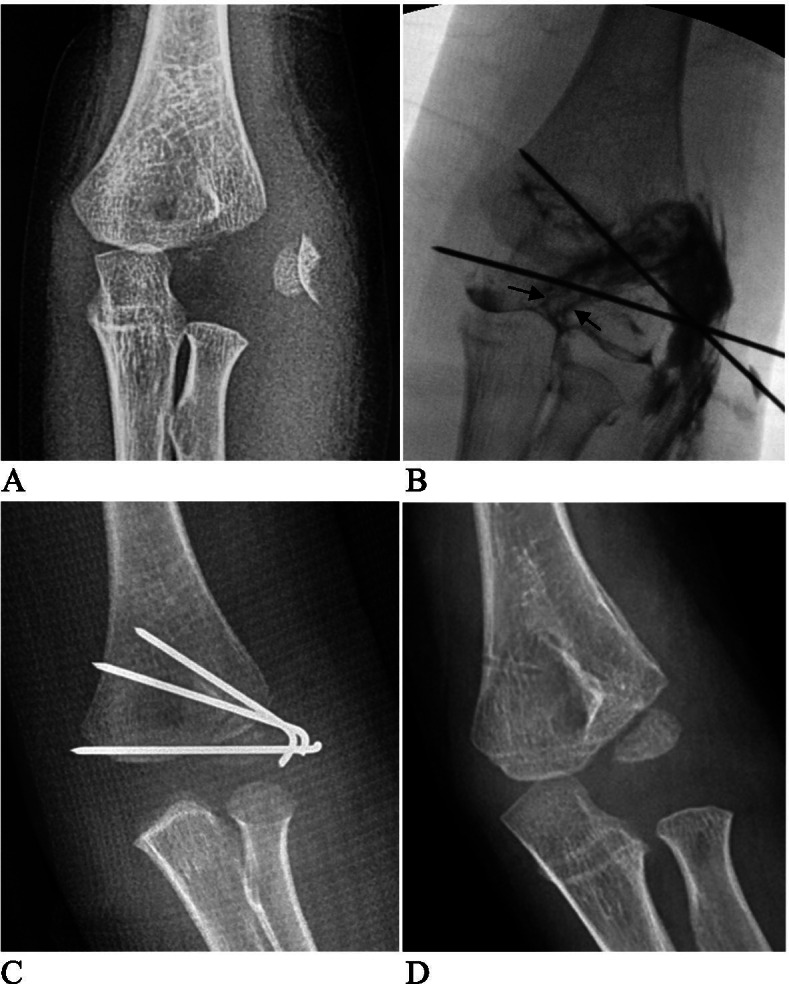
A Song stage 5 and Milch typeILCHF of a 5 years old boy. **A** Pre-operative anterior-posterior X-ray. **B** Intro-operative arthrogram after CRPP showed unacceptable fracture gap (>2 mm, black arrows), then ORIF was performed. **C** X-ray post ORIF. **D** X-ray 6 months post ORIF after internal pins’ removal showed a nearly normal bone structure

Although the use of CRPP in treating LCHFs displaced by > 4 mm has achieved promising results for more than a decade, CRPP has not been widely adopted. Technical difficulty might be the main reason for the unpopularity of CRPP in treating LCHFs displaced by > 4 mm. Most studies have reported favorable results where CRPP has been used to treat LCHFs with displacement between 2 mm and 4 mm, with a low success rate in cases with greater displacement, especially in total displacement [7, 13, 14]. Ramo et al. validated the high intra-observer and inter-observer reliability of the Song classification. They achieved an 89.5% (51/57) success rate with CRPP in Song stage 4 LCHFs. However, they failed to replicate the success of CRPP in the Song stage 5 LCHFs [14]. In our previous work, the first 10 consecutive attempts of CRPP failed before we became skilled at performing CRPP for Song stage 4 and 5 LCHFs. Later, we achieved an approximate 75% CRPP success rate, even in Song stage 5 cases [15]. In the present study, we achieved an overall CRPP success rate of 78% (45/58). Another reason for the lack of acceptance of CRPP is the concern that CRPP achieves imperfect articular reduction, leading to potential complications such as malunion, delayed union, growth disturbance, and future surgery. Because LCHFs are intra-articular fractures, most surgeons can only accept anatomical reduction. Accordingly, ORIF may be the safest treatment option [7, 9, 10]. Justus et al. compared CRPP and open reduction and percutaneous pinning (ORPP) in treating LCHFs and reported that there was no need to change the postoperative treatment for CRPP even in totally displaced cases. They found no difference in complications between CRPP and ORPP [13]. In the present case, we found no differences in pre- and post-operative displacement. Moreover, there were no differences in complications between CRPP group and ORIF group during the follow up period. Furthermore, our results showed that changing treatment from CRPP to ORIF did not affect prognosis (Fig. 2). In addition, complications such as malunion or growth disturbance usually occur in cases with unstable fixation or delayed presentation [18, 19]. Therefore, concerns of inadequate fracture reduction, malunion and growth disturbance may be unnecessary.

Justus et al. classified LCHFs as high-grade displaced LCHFs (Song stages 4 or 5), and low-grade displaced LCHFs (Song stages 2 or 3). They concluded that high-grade displaced LCHFs were more likely to be treated with open reduction [13]. This is true when comparing high-grade LCHFs to low-grade LCHFs. However, this is not the case in Song stage 4 versus 5 LCHFs. The present study showed that there was no difference in the success rate of CRPP between Song stage 4 and 5 LCHFs, but there was a difference between Milch type I and type II LCHFs. A Milch type I fracture was more likely to be treated with ORIF, while a Milch type II LCHF was more likely to be treated with CRPP. This suggested that the Milch type of LCHFs, and not the pre-operative displacement, was the key factor in the success of CRPP in LCHFs displaced by > 4 mm.

Growth arrest and fishtail deformity were not found in the present study at a mean follow-up period of 13.9 months. This was in accordance with previous studies. The studies by Song et al. reported no growth disturbance at an average follow-up of 25 months and 30 months, respectively [11, 12]. Ramo et al. and Justus et al. also reported no growth disturbance at a follow-up period less than 6 months [13, 14]. However, the study by Cates et al. showed that the average time from injury to capitellar ossification center growth arrest was 2.6 years [20].

**Fig. 3:**
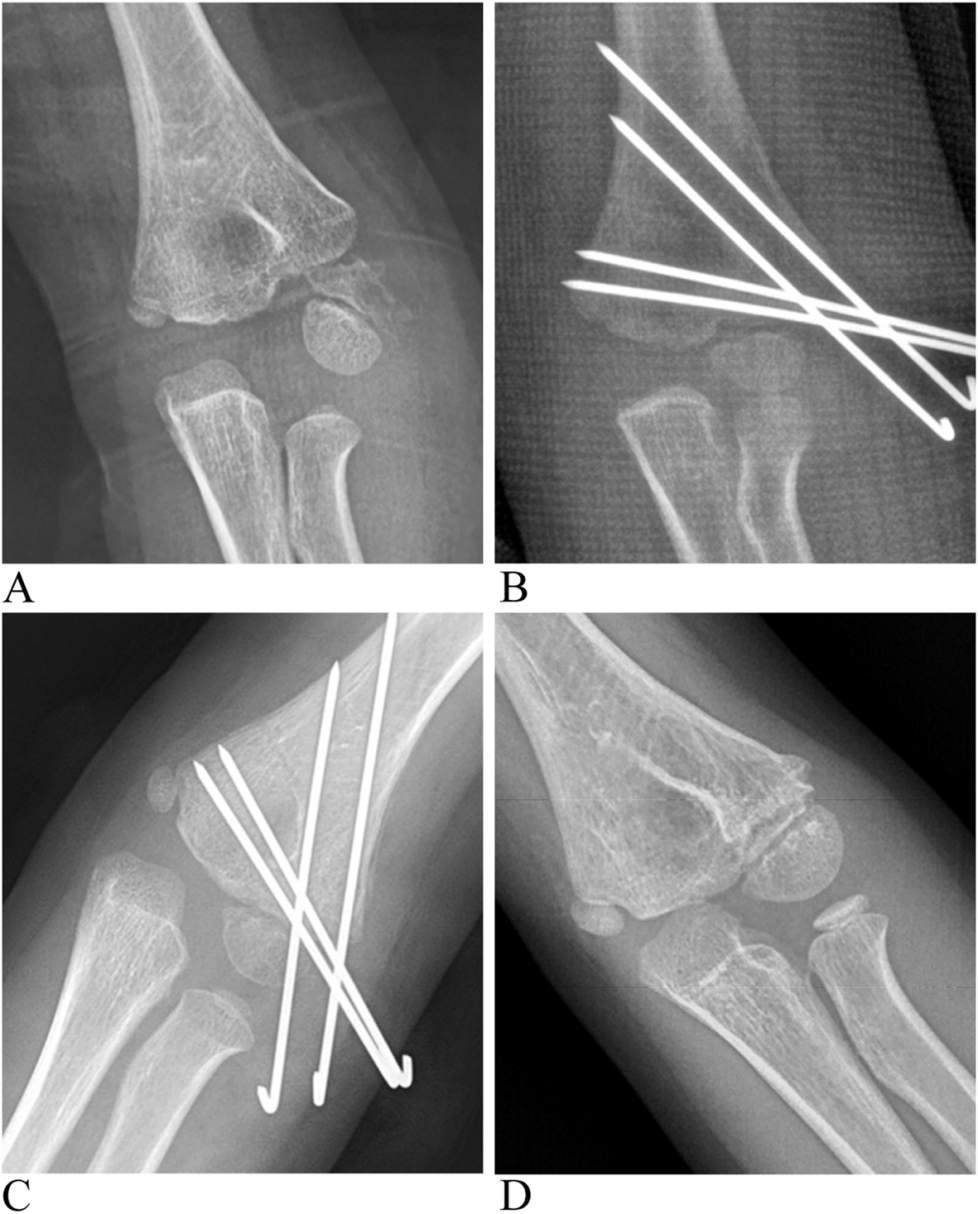
A 5 years old girl with a Song stage 5 and Milch type II LCHF treated with CRPP. **A** Pre-operative anterior-posterior X-ray. **B** Post-operative X-ray showed satisfactory reduction. **C** X-ray 4 weeks post surgery showed well union of fracture. **D** X-ray 16 months post surgery showed a decreased Baumann angle

**Fig. 4:**
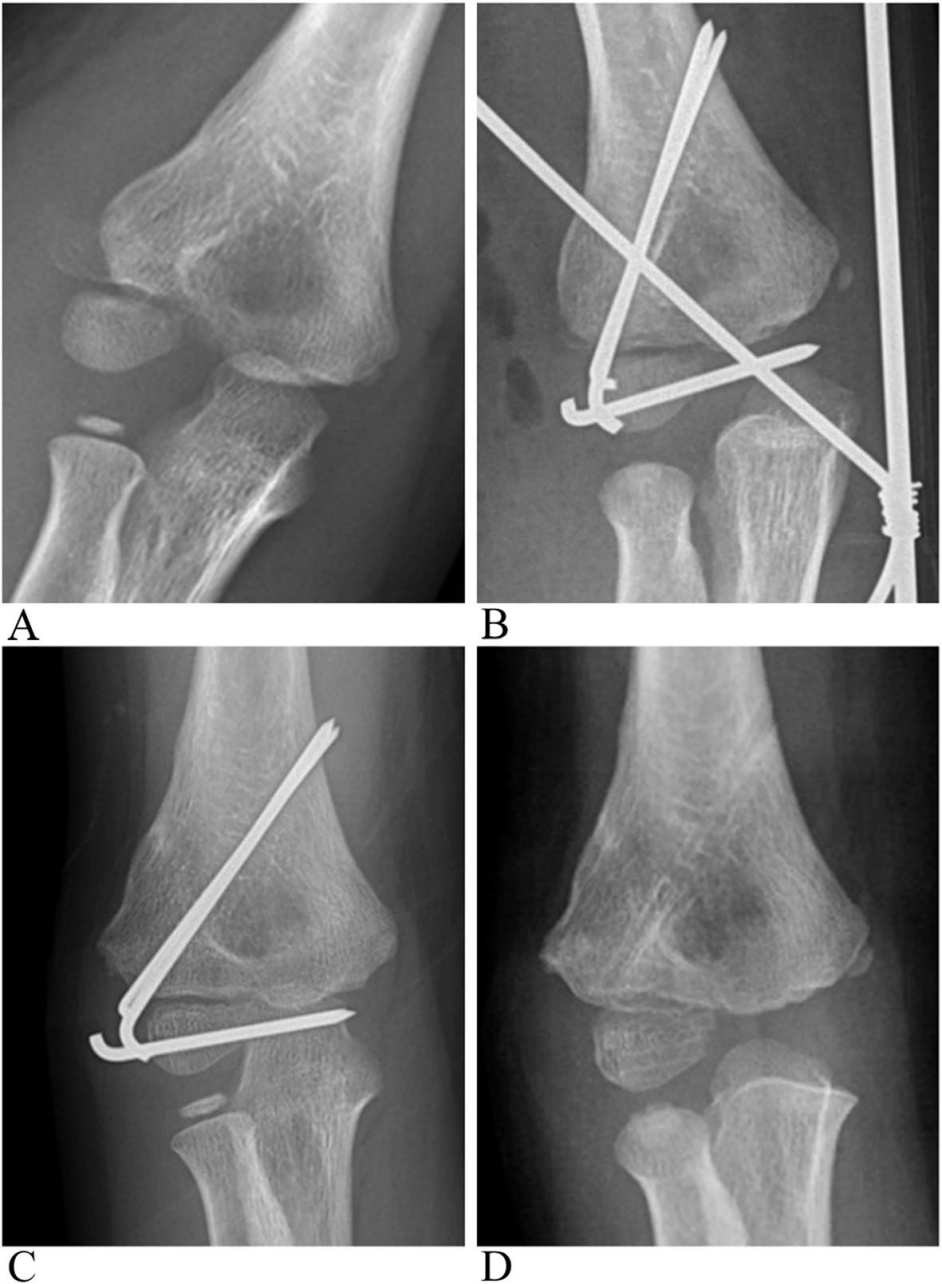
A 5 years old boy with a Song stage 4 and Milch typeILCHF treated with ORIF directly. **A** Pre-operative anterior-posterior X-ray. **B** post-operative X-ray showed nearly anatomic reduction. **C** 5 months post operation showed well fracture union but a decreased Baumann angle. **D** X-rays after internal pins’ removal

A decreased carrying angle was common complication in both CRPP and ORIF groups. This usually does not cause obvious cubitus varus but often leads to a straight elbow. This might be due partly to a residual lateral open wedge gap or tilt of distal fragment after reduction (Fig. 3), leading to a decreased Baumann angle, which is an important index for the carrying angle. Another reason for this might be overgrowth of lateral condyle of humerus (Fig. 4).

Bone spurs and lateral prominences were also common complications in all three groups in the present study. Pribaz et al. found that lateral bone spurs correlate with initial displacement and surgical treatment, with bone spur size associated with the amount of initial fracture displacement. However, lateral bone spurs are usually asymptomatic and do not influence clinical outcomes [21]. In our experience, the formation of lateral prominences is related to periosteal tearing and lifting, which cannot be anatomically repositioned during fracture reduction and soft tissue repair. Thus, new bone fills the gap between the lateral cortex and periosteum to form a bone spur.

Obtaining a functional elbow is the goal of treatment regardless of the type of procedure performed. In the present study, the average follow-up time was 13.9 months, and the results showed that both CRPP and ORIF groups achieved satisfactory range of motion (ROM) and elbow function. This is in accordance with Bernthal et al.’s results, who found no difference in ROM between surgical treatment and conservative treatment at 18 weeks after injury, and no difference in ROM between CRPP and ORIF cases [22].

The present study has several limitations including the intrinsic limitations of a retrospective study, small sample size, and short follow-up period. Therefore, a prospective randomized control study with more cases and longer follow-up is necessary. Moreover, more efforts should be made to study the mechanism and anatomical basis of CRPP in LCHFs with displacement > 4 mm.

## Conclusions

Both CRPP and ORIF can achieve satisfactory clinical outcomes in treating LCHFs displaced by > 4 mm. No difference was found in the complications and prognosis between the two groups. However, CRPP shows some advantages over ORIF, like less invasive surgery, no obvious scarring, and no need for secondary surgery with anesthesia for pin removal.

## Data Availability

The data and materials used in the current study are available from the corresponding author upon reasonable request.

## Abbreviations

LCHFs: Lateral condylar humerus fractures
CRPP: Closed reduction and percutaneous pinning
ORIF: Open reduction and internal fixation
ORPP: Open reduction and percutaneous pinning
ROM: Range of motion
